# Nutritional Value of Whole Corn Germ Obtained by the Wet Method for Poultry

**DOI:** 10.3390/vetsci12050470

**Published:** 2025-05-14

**Authors:** Michele Bernardino de Lima, Carlos Bôa-Viagem Rabello, Gabriel Henrique Nacamura da Silva, Jaqueline Aparecida Pavanini, Rita Brito Vieira, Isidro Chemane Argentina, Lizandra Amoroso, Edney Pereira da Silva

**Affiliations:** 1 Department of Animal Production and Health, School of Veterinary Medicine, UNESP—São Paulo State University, Araçatuba 16050-680, Brazil; michele.bernardino@unesp.br; 2 Department of Animal Sciences, Rural Federal University of Pernambuco, Recife 52171-900, Brazil; carlos.rabello@ufrpe.br; 3 Department of Animal Sciences, College of Agriculture and Veterinary Sciences, UNESP—São Paulo State University, Jaboticabal 14884-900, Brazil; gabriel.nacamura@unesp.br (G.H.N.d.S.); j.pavanini@unesp.br (J.A.P.); rita.brito@unesp.br (R.B.V.); isidro.a.chemane@unesp.br (I.C.A.); lizandra.amoroso@unesp.br (L.A.); 4 Departamento da Agricultura, Escola Superior de Negócios e Empreendedorismo de Chibuto (UEM-ESNEC), Universidade Eduardo Mondlane, Nas Esquinas das Avenidas Samora Machel e 25 de Setembro, Chibuto, Gaza C.P. 63, Mozambique

**Keywords:** amino acid digestibility, corn co-product, fatty acid digestibility, metabolizable energy, roosters

## Abstract

This study aimed to assess the nutritional value of whole corn germ (WCG) in the diet of chickens. Amino acid digestibility, fatty acid digestibility, and metabolizable energy were evaluated. A metabolism assay was conducted using the precise feeding method on roosters. A completely randomized design was used, with two treatments and ten replications per rooster in each experimental unit. The treatments were as follows: WCG1, precise feeding with WCG; and WCG2, fasting birds to determine metabolic and endogenous losses in energy and fat. The variables analyzed were coefficients for amino acids in corn germ meal and fatty acid digestibility. The results showed that the standardized digestibility coefficients for amino acids in corn germ meal were (in g/kg) as follows: Lys, 920; Thr, 780; Met + Cys, 800; Arg, 910; His, 890; Ile, 950; Leu, 970; Phe + Tyr, 870; Val, 980; Gly + Ser, 740; Ala, 960; Asp, 870; and Glu, 930. The average fatty acid digestibility and AMEn were 850 g/kg and 4934 kcal/kg, respectively. Corn germ meal, which showed high digestibility of nutrients and energy, is considered an interesting ingredient for diets requiring high energy concentration.

## Simple Summary

This study assessed the nutritional value of corn germ meal in chicken diets, emphasizing its amino acid and fatty acid digestibility and metabolizable energy. The results demonstrated that corn germ meal possesses high digestibility for amino acids and fatty acids and a high energy content. These findings suggest that corn germ meal is a valuable ingredient for poultry diets, particularly for those requiring enhanced energy concentrations and nutrient-efficient utilization.

## 1. Introduction

Digestible amino acids have been included in feed formulations to provide low-cost and high-performance diets for birds. The whole corn germ (WGM) is an alternative feed ingredient that can be obtained through the wet degermination of the corn grain without undergoing the lipid (corn oil) extraction process [1,2,3,4], and yields approximately 130 g/kg of the grain [1,4,5].

Previous studies [4,5] have shown that the chemical compositions of crude protein (100–115 g/kg), ether extract (500–560 g/kg), gross energy (7000–7243 kcal/kg), and mineral matter (19–50 g/kg) are relevant to be used in broiler’s feed. However, the results of Lima et al. [4] and Albuquerque et al. [5] for the energy values are divergent. Lima et al. [4] observed that the digestible energy of corn germ meal increased by 13 kcal kg^−1^ per day in the diet of 40-day-old broiler chickens. When compared to corn, WCG contains 79.5% less glycine and serine, 38.51% less leucine, 16.95% less methionine and cystine, 16.8% less phenylalanine and tyrosine, and 9.7% less isoleucine, according to [6].

In turn, the study of Albuquerque et al. [5] showed that an increase of 200 to 300 g/kg of WCG in the reference diet did not affect the value of nitrogen-corrected apparent metabolizable energy (AMEn). The average AMEn determined by these authors for laying hens was 4548 kcal/kg. Despite the difference between digestible energy [4] and metabolizable energy [5], the specific energy in laying hens is expected to be higher than that in broiler chickens, as an effect of age on the energy use [1,4]. The variations in the results reported across studies may be attributed to several factors, including the type of processing applied to the ingredient (e.g., wet or dry methods), the physiological stage of the birds, the analytical methodologies employed, and the inherent chemical composition of the feed ingredient. Therefore, additional studies are needed to establish the energy value of WCG. Although some studies have focused on the WCG obtained using the dry processing (e.g., [7,8,9]), their results cannot be extrapolated to the WCG obtained using the wet method because of differences in the chemical composition and the effects on the use of energy, as observed by [4,5].

To the best of our knowledge, there is no information in the literature on the amino acid digestibility coefficients of the WCG obtained by wet processing. Despite WCG having over 500 g/kg of fat in the dry matter and a high digestibility value [4], there is also no information on the profile of fatty acids nor on their digestibility coefficients for birds. Thus, the aim of this study was to determine amino acid digestibility, fatty acid digestibility, and the metabolizable energy values of WCG using cecectomized roosters.

## 2. Materials and Methods

### 2.1. Animals, Management, and Experimental Design

The experiment was conducted in the Poultry Laboratory of the Department of Animal Science of the Federal University of Viçosa. Twenty 36-week-old Leghorn cecectomized roosters with an average weight of 1840 ± 181 g were used. The roosters were individually placed in metabolic cages, which were already adapted to the installations. The average temperature recorded was 24 ± 2 °C. A completely randomized design was used, with 2 treatments and 10 replications per rooster in each experimental unit. The treatments were as follows: WCG1, precise feeding with WCG; and WCG2, fasting birds to determine metabolic and endogenous losses in energy and fat. The whole corn germ used in this research presented the following chemical composition: 954.2 g/kg of dry matter, 111.0 g/kg of crude protein, 564.8 g/kg of ether extract, 53.5 g/kg of crude fiber and 50.0 g/kg of mineral matter, calcium 4.5 g/kg, phosphorus 16.0 g/kg, and 7039 kcal kg^−1^ of gross energy. The composition of amino acids content is presented in Table 1.

### 2.2. Assay for True Metabolizable Energy

The animals were kept in a solid fasting regimen for 36 h for the complete emptying of the gastrointestinal tract. Then, each rooster received precisely 30 g of WCG via a funnel probe directly into the crop [11]. Feeding was divided into two times of 15 g, at 8 h and 16 h after the beginning of the experiment, to prevent regurgitation. Excreta collection started after the first supply of feed and lasted 56 h. The collected material was weighed and stored in a freezer at −20 °C. At the end of the experiments, the material was defrosted, homogenized, placed in a freezer for 14 h at −80 °C, and then lyophilized for a period of 48 h.

### 2.3. Laboratory Analyses

Afterwards, feed and excreta samples were grinded and analyzed for dry matter (DM), nitrogen (N), gross energy (GE), amino acid composition, and fatty acid composition.

The Kjeldahl method described by the Association of Official Analytical Chemists—AOAC [12] was used to determine the protein content. This method quantifies the total nitrogen content, which is converted to protein by multiplying it by a conversion factor of 6.25. Total nitrogen was obtained by digestion of the samples using a digestion block (model Tecnal, TE-007A, Piracicaba, Brazil). Distillation was carried out in a nitrogen still (Tecnal, TE-036/1, Piracicaba, Brazil) using sodium hydroxide and boric acid solutions, and titration was subsequently carried out with hydrochloric acid solutions.

#### 2.3.1. Analytical Standards

##### Amino Acid Analysis

To determine the amino acid composition, samples were hydrolyzed with 6N hydrochloric acid for 24 h at 110 °C under a nitrogen atmosphere. Amino acids that were released by acid hydrolysis and reacted with phenyl isothiocyanate were separated using liquid chromatography model ALC 204 (Waters), equipped with two M6000A solvent delivery systems (Waters, Milford, MA, USA), controlled by an M660 gradient programmer. Detection was performed by an M440 fixed wavelength spectrophotometer (254 nm/0.2 AUFS). Samples were injected by an M712 WISP automatic injector (Waters, Milford, EUA). Separation was performed on a Pico-Tag analysis column (Waters) (3.9 × 150 mm, stainless steel), maintained at 40 °C with a column heater (Waters, Milford, EUA), described by [13]. Quantification was conducted by multilevel internal calibration using alpha-aminobutyric acid as the internal standard.

##### Fatty Acid Analysis

The fatty acid profile was analyzed using gas chromatography of methyl esters, following the AOAC method Ce 1–91 [12]. This method was performed using a gas chromatograph (Shimadzu GC-2030, Kyoto, Japão) equipped with a flame ionization detector (FID). This equipment contains a Split/Splitless injector and a highly polar fused silica capillary chromatographic column (CP-Sil 88, Santa Clara, CA, USA) 100 m long and 0.25 mm wide. The extraction gas was hydrogen. The samples were injected directly using fatty acid methyl esters (FAMEs), and the working temperatures were 200 °C in the injection, 240 °C in the column (at a speed of 20 °C/min), and 250 °C in the FID, according to [14] the test samples with the retention time of methyl esters’ standard chromatography. Quantification was conducted by converting the peaks of area percentage to mass percentage.

### 2.4. Variables and Calculations

To determine the metabolizable coefficients of amino acids and fatty acids in WCG, the individual values of the feed intake and excretion of each replicate were used. To calculate the standardized amino acid digestibility (SAD), an average of ten replicates per amino acid or fatty acid of the basal metabolic and endogenous losses were considered.

Only the amino acids were calculated using the SAD values of basal metabolic and endogenous losses, obtained by [10] and established with a free-nitrogen or protein diet (NFD), hydrolyzed casein or highly digestible protein (HPD), and fasted birds. This calculation was also used to generate the mean value of basal loss; thus, SAD values were generated for each amino acid.

For the analysis of fatty acids, the values obtained from the excreta of fasted birds were used. The variables analyzed were the SAD, apparent metabolizable energy (AME), true metabolizable energy (TME), nitrogen-corrected true metabolizable energy (TMEn), and the metabolizable coefficients of gross energy (MCGE) of WCG in relation to AMEn (MCGE = AMEn/GE × 100) and to TMEn (MCGE = TMEn/GE × 100).

### 2.5. Analysis Statistics

The statistical model for the random design with k treatments in which *p* variables are measured is the following: y_ij_ = μ_r_ + τ_ir_ + ε_ir_ i = 1, …, k; r = 1, …, *p*; where: r = indexer of the variables; y_ir_ = observation r-^th^ variable under the effect of the i-^th^ treatment; µ_r_ = the overall mean the r-^th^ variable; τ_ir_ = the fixed effect of treatment i under r-^th^ variable; ε_ir_ = random error with mean 0 and variance σ^2^. The SAD data were subjected to a multivariate exploratory analysis, using a factorial analysis to obtain the relationships contained in the variable group, which included 14 analyzed amino acids, to be explained using a reduced number of new variables. The factors were extracted using a principal component analysis computed from the correlation matrix between variables. Two factors were extracted; the first linear combination of original variables represented the maximum possible variability contained in the sample; the second factor was responsible for much of the remaining variability. The factors were standardized and dimensionless variables (μ = 0, σ^2^ = 1). The effect of the correction of the metabolic and endogenous basal losses on the SAD of each amino acid was tested using a multivariate analysis of variance (MANOVA), considering the statistical model: Y = XΒ + ε, where Y is the matrix of dimensional observations; X is the matrix of the dimension design; Β is the matrix of the dimension parameter; and ε is the matrix of the error of dimensions. The significant differences between the SAD data provided by the various corrections for endogenous basal loss were compared using a Duncan’s test.

## 3. Results

The crude protein content of WCG was 111 g/kg and of this total, and the amount of essential amino acids (EAAs) (Lys, Thr, Met, Arg, His, Ile, Leu, Phe, and Val) corresponded to approximately 40% of the total amino acids and 60% of the non-essential amino acids (NEAAs). The main components of the WCG proteins were Leu (76 g/kg), Arg (63 g/kg), Val (55 g/kg), Glu (6.3 g/kg), Gly (46 g/kg), Ser (37 g/kg), and Asp (35 g/kg) among the analyzed NEAA (Cys, Tyr, Gly, Ser, Ala, Asp, and Glu).

The SAD values and the endogenous losses determined in this study and in [10] showed a correlation coefficient of 0.8 when Gly and Gly + Ser were excluded from the calculation. When Gly and Gly + Ser were included, the coefficient value was 0.4 (Table 1). The EAA and NEAA of SAD were the following: 880 and 840, 900 and 970, 900 and 980, and 790 and 820 g/kg for the fasting methods (present study), and NFD [10], HPD [10], and the literature fasting methods [10], respectively (Table 1).

The factor analysis allowed the extraction of the relationships contained in the SAD calculation on the basis of four procedures for the correction of endogenous losses in amino acids, synthesizing the sample space of 17 amino acids to two factors and retaining 95.5% of the original variance of the data (Table 2). The first factor explained approximately 84% of the variance, whereas 11% of the variance was explained by the second factor. The amino acids Thr (0.59), Cys (0.39), Tyr (0.18), and His (0.09) showed a low correlation with the first factor and a high correlation (over 0.8) with the second factor (Table 2).

The results obtained by the multiple comparison analysis (Table 3) applied to the extracted factors showed that the calculated SAD was similar to the NFD and HPD; on the other hand, the SADs based on the endogenous losses in fasted birds determined in this study and obtained from the literature [10] differ among themselves and from the other techniques (NFD and HPD).

**Figure 1:**
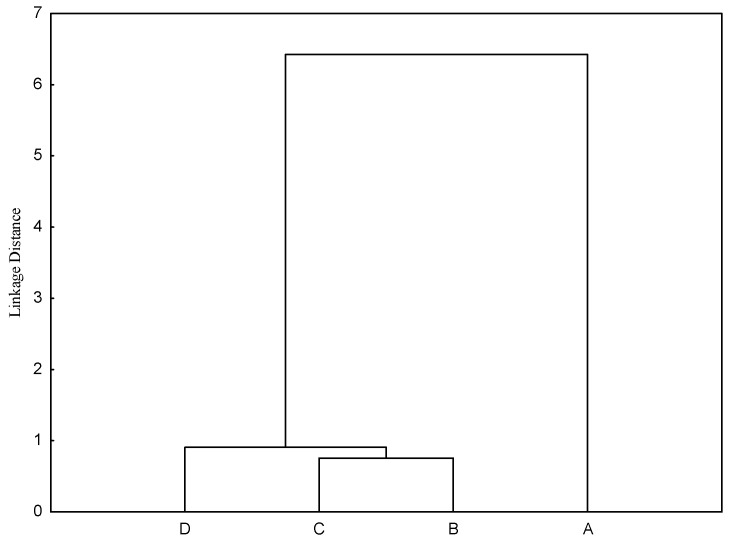
Cluster analysis in order to classify methodology used correction of endogenous losses in amino acids, based on Euclidean distances. A—Fasted present study; B—Nitrogen-free diet (NFD), obtained by [11]; C—Highly digestible protein (HDP), obtained by [11]; D—Fasted, obtained by [11].

Although the individual values of CSM were correlated, there was a difference in the average value of each amino acid lost in the endogenous flow, according to the results of the clustering analysis (Figure 1). This type of analysis allows an interpretation using Euclidian distance, i.e., the closest similarity has the smallest distance. In the present study, the NFD and HDP had the smallest distance (0.75). On the other hand, when birds were submitted to fasting, the largest distance (6.43) was found between results obtained by the same method (Figure 1). An individual analysis showed that the greatest absolute obtained distance was between the NFD and fasting birds (6.81), followed by the HDP (6.73). When the same comparison was made by [10], the distances between fasting birds and the NFD and fasting birds and the HDP were 0.91 and 1.09, respectively.

The fatty acid profiles of the WCG, excreta, and endogenous losses were determined, and the apparent and true digestibility of fatty acids was calculated (Table 4). The main fatty acid contents determined in the WCG were linolenic acid C18:3n3, 6.0 g/kg; palmitic acid C16:0, 174 g/kg; oleic acid C18:1, 302 g/kg; and linoleic acid C18:2 519 g/kg. The apparent and true digestibility values of fatty acids were 840 and 850 g/kg, respectively.

The correction for the nitrogen balance in the metabolizable energy system increased the apparent value in 302 kcal/kg. The difference between the TMEn and AMEn was 6.6%, and the difference between the TME and AME was over 22.6%. The metabolizable gross energy coefficients regarding the apparent and true systems were 59% and 70%, respectively (Table 5).

## 4. Discussion

It is noteworthy that the WCG of the present study differs from those presented in the literature [6,7,8]. The analyzed WCG has six times more ether extract (EE) than the reported germs in the main recommendation tables [6,15,16]. Even though over half of the WCG dry matter is composed of fatty acids, the proportion of EAA and NEAA did not change when compared to the germ obtained through the dry method [6,15,16]. Despite this difference, the composition of the total amino acids was similar; however, for some amino acids, such as the first limiting amino acids for poultry (Met, Lys, Thr, Val, Ile, and Arg), the coefficients of digestibility and digestible amino acids levels were higher than those reported in the literature of the corn germ meal obtained by the dry method [6,15,16].

The WCG obtained by the wet method showed a change in the profile of EAA. There was an increase in the concentration of valine and a decrease in leucine when compared to the same base (g by 100 g CP). Leucine is an amino acid rich in ingredients, and it competes with valine and leucine for the absorption site; thus, the WCG can be used as a strategy when the goal is to reduce dietary leucine. In the present study, an exercise to calculate the SAD with the values of the endogenous amino acid losses determined in this study and in others presented in the literature was carried out.

Based on the results presented herein, it was not possible to reach a consensus on the use of the endogenous losses in fasted animals because of the differences in the SAD of amino acids (Table 3). This finding is in contrast with the initial hypothesis that methodological similarities would provide similar values. However, the similarities or Euclidean distances were lower for the NFD and HPD methods, corroborating the results of [10]; this endorses the use of the endogenous loss value based on the NFD because of its simplicity and the fact that the results can be reproduced with this method.

The results obtained for Gly and Gly + Ser do not support the use of the endogenous loss values determined with the NFD, because the SAD of these amino acids exceeded 100% when values from [10] were used, regardless of the method. Because of the simplicity of the applied methodology, it was not possible to derive a clear explanation. However, based on the function of Gly, a hypothesis would relate to the endogenous loss in Gly with the total excreted uric acid [17]. For this reason, further studies on endogenous loss values are necessary so that these values can be widely used or so that these values can be widely utilized, or so that methodologies can be refined to minimize limitations.

The methodology used to explore the relationships contained in the SAD was calculated using four procedures for the correction of endogenous losses in amino acids, and was shown to be sustainable, allowing the reduction in sample space without the loss in the power of inference. The results of the multiple comparisons using the factors were like the conclusions reached by [10] when each amino acid was analyzed individually.

Approximately 830 g/kg of the fatty acids contained in the EE of WCG is essential in the diet of birds. Some studies have shown that unsaturated fatty acids can reduce abdominal fat and total body fat by active β-oxidation, in contrast to saturated fats, according to Fouad and El-Senousey [18]. This fatty acid profile is important in the nutrition of broiler chickens and commercial layers, because of these animals’ susceptibility to liver problems. To benefit from the WCG fatty acid profile, the digestibility coefficients of these fatty acids should be considered. According to the results presented here, 150 g/kg total fatty acid content was not absorbed; this result may aid in establishing the levels of fatty acids in the diet formulation. The digestibility of fatty acids in feed ingredients is not commonly determined. The most advanced recommendation tables only separate lipid materials into fatty acids and non-fatty acids [16]. Therefore, because of this lack of information, we were unable to compare our data with the literature data.

The proximity of the apparent and true digestibility coefficients was attributed to the small contribution of the endogenous fatty acids analyzed. For metabolizable energy, the apparent and real systems were different, in which the TMEn was 22% higher than the AMEn. Dale and Fuller [19], while comparing the energy systems, commented that the TMEn values are an average of 14% higher than those determined for the AMEn. These values were more similar when the correction to the nitrogen balance was applied, reducing the difference to 7%; this probably occurred because the birds were in a negative nitrogen balance.

The energy values of corn germ were determined by using force-feeding with roosters [7], and the following values for the dry matter of AME, TME, AMEn, and TMEn, respectively, were found: 3134 kcal/kg, 3945 kcal/kg, 3509 kcal/kg, and 3782 kcal/kg. Another study using whole corn germ and the force-feeding method with roosters [8] found values of 3747 and 4094 kcal kg^−1^ for natural matter of AMEn and TMEn, respectively. It is noteworthy that these results are based on the product’s fat content, indicating that the difference in processing reflects the nutritional quality, since the existing fat content in this study was over 50% of its dry matter. The authors mentioned [8] reported an EE value 82.3% lower than that used to generate the data of this study.

The WCG was evaluated (565 g/kg of fat content) for broilers at different ages [1], and the authors found values of 4228, 4413, 4620, 4846, and 5095 kcal/kg of natural matter for AMEn in 7, 14, 21, 28, and 35-day-old broilers, respectively. Among the published studies, these values are the most similar to the results of the present study. Our results, therefore, confirm the values found by [1,4], which were similar to the values found for broilers of older age, possibly because of these animals’ intestinal development and their ability to digest and absorb lipids. The use of agro-industrial by-products in animal nutrition contributes positively to the sustainability of production systems and may serve as an effective strategy to reduce feed costs. With the continued growth of the corn industry across the Americas, the generation of by-products is expected to increase, offering a potential alternative to conventional feed ingredients. However, a thorough understanding of the chemical composition, energy content, availability, and cost-effectiveness of these by-products is essential. Such information enables nutritionists to formulate more precise and economically viable diets, optimizing both animal performance and production efficiency.

## 5. Conclusions

The coefficients’ standardized digestibility of amino acids of whole corn germ was high for most essential amino acids. These findings suggest that this by-product is a promising source that can significantly contribute to balanced nutritional formulations. The average digestibility of fatty acids in WCG was 850 g/kg. The corrected metabolizable energy od WCG was 4934 kcal/kg. The use of the multivariate exploratory analysis allowed the reduction of sample space without compromising inferential power.

## Figures and Tables

**Table 1:** Total amino acid content and standardized amino acid digestibility calculated considering different basis endogenous basal loss bases of the whole corn germ with 11.1% CP (as-is basis, g/kg).

Amino Acids	Total	Fasted	NFD ^II^	HDP ^III^	Fasted ^IV^	Average ± SEM ^V^
		Present Study ^I^	[10]			
Lys	5.1	910	920	940	820	900 ± 27
Thr	4.1	770	780	770	610	730 ± 41
Met	2.1	870	870	880	810	860 ± 16
Cys	1.8	780	710	680	530	670 ± 53
Met + Cys	3.9	830	800	790	680	780 ± 32
Arg	7.0	990	910	890	820	900 ± 37
His	3.9	960	890	890	840	890 ± 25
Ile	3.6	840	950	1010	790	900 ± 49
Leu	8.4	890	970	950	850	920 ± 27
Phe	4.6	800	820	790	700	780 ± 28
Tyr	3.1	850	910	880	730	840 ± 40
Phe + Tyr	7.7	820	870	840	710	810 ± 33
Val	6.1	870	980	980	830	920 ± 38
Gly	5.1	640	1570	1550	1380	1290 ± 22
Ser	4.1	850	860	940	710	840 ± 48
Gly + Ser	2.1	740	1220	1240	1050	1060 ± 115
Ala	1.8	860	960	950	830	900 ± 32
Asp	3.9	800	870	860	710	810 ± 37
Glu	7.0	870	930	990	830	91 ± 3.53

^I^ Average represents 10 replicates with 1 bird per replicate; ^II^ NFD, nitrogen-free diet; ^III^ HDP, highly digestible protein; ^IV^ average represents four replicates with one bird per replicate; ^V^ standard error mean.

**Table 2:** Correlation matrix of the factor analysis for 14 amino acids that compose the corn germ meal ^I^.

Amino Acids	Factor 1	Factor 2
Lys	0.69	0.72
Thr	0.59	0.80
Met	0.63	0.76
Cys	0.39	0.91
Arg	0.87	0.45
His	0.09	0.99
Ile	0.76	0.64
Leu	0.83	0.46
Phe	0.93	−0.18
Tyr	0.18	0.97
Val	0.83	0.54
Gly	0.64	0.69
Ser	0.71	0.66
Ala	0.88	0.41
Eigenvalue	14.22	2.02
% Total variance	83.66	11.86
Cumulative eigenvalue	14.22	16.24
Cumulative %	83.66	95.52

^I^ Extraction method: principal component analysis. Rotation method: Varimax raw.

**Table 3:** Multiple comparison analysis applied to the extracted factors of corn germ meal.

Source Endogenous Amino Acid	Factor 1	Factor 2
^I^ Fasted—Present study	−1.13 ^c^	1.21 ^a^
^II^ NFD—[11]	0.64 ^a^	−0.09 ^b^
^III^ HDP—[11]	0.84 ^a^	−0.27 ^b^
^IV^ Fasted—[11]	−0.34 ^b^	−0.85 ^bc^
F	21.7	16.5
*p* value	<0.0001	<0.0001

Means followed by the same letters in the same column do not indicate statistical differences (*p* > 0.05) according to the Duncan’s test.; ^I^ average represents 10 replicates with 1 bird per replicate; ^II^ NFD, nitrogen-free diet; ^III^ HDP, highly digestible protein; ^IV^ average represents 4 replicates with 1 bird per replicate.

**Table 4:** Content of total fatty acids, endogenous fatty acid, apparent and true digestibility coefficients of corn germ meal (as-is basis, g/kg).

**Fatty Acids**	**Total** **Fatty Acids**	**Endogenous** **Fatty Acid**	**Apparent Digestibility Coefficients**	**True Digestibility Coefficients**
Palmitic C16:0	174.0	283.0	893.0	900.0
Oleic C18:1	302.0	207.0	796.0	799.0
Linoleic C18:2	519.0	172.0	885.0	887.0
Linolenic C18:3n3	6.0	8.0	80.4	810.0
Stearic C18:0	0.0	166.0	0	0
y-Linolenic C18:3n6	0.0	22.0	0	0

**Table 5:** Apparent metabolizable energy values (AME, kcal/kg), nitrogen-corrected apparent metabolizable energy (AMEn, kcal/kg), true (TME, kcal/kg), nitrogen-corrected true metabolizable energy (TMEn, kcal/kg), metabolizable gross energy coefficient (MGEC, %), and corn germ meal (as-is basis).

Corn Germ Meal	AME	AMEn	TME	TMEn	MGEC	
					GE/AMEn	GE/TMEn
Average	4632	4934	5683	5261	66.58	71.00
Standard error mean	0.066	0.048	0.102	0.090	0.65	1.21

GE, gross energy.

## Data Availability

The original contributions presented in this study are included in the article. Further inquiries can be directed to the corresponding author.

## References

[1] Lopes E.C., Rabello C.B.V., Macambira G.M., Santos M.J.B.D., Lopes C.C., Oliveira C.R.C.D., Silva J.D.C.R.D., Silva B.A., Nascimento J.C.S., Ribeiro A.G. (2024). Effect of different levels of whole corn germ on energy values and ileal digestibility in broilers. An. Acad. Bras. Ciências.

[2] El-Shenawy A.M., Tahoon A.E.-N.Y. (2024). Impact of Dietary Corn Germ Meal Inclusion and Bile Acids Supplementation on Growth Performance and Health Status of Japanese Quail. Egypt. J. Anim. Health.

[3] El-Katcha M.I., Soltan M.A., Shewita R.S., Abdel Naby A.M., Alagawany M., Azzam M.M., Lestingi A., El-Naggar K. (2024). Use of corn germ meal and bile acids in laying quail diets: Effect on production, reproduction, health and economics. Poult. Sci..

[4] Lima M.B., Rabello C.B.-V., Silva E.P.D., Lima R.B., Arruda E.M.F.D., Albino L.F.T. (2012). Effect of broiler chicken age on ileal digestibility of corn germ meal. Acta Sci. Anim. Sci..

[5] Albuquerque C., Rabello C., Santos M., Lima M., Silva E., Lima T., Ventura D., Dutra W. (2014). Chemical composition and metabolizable energy values of corn germ meal obtained by wet milling for layers. Rev. Bras. Ciência Avícola.

[6] Rostagno H.S., Albino L.F.T., Calderano A.A., Hannas M.I., Sakomura N.K., Perazzo F.G., Rocha G.C., Saraiva A., Abreu M.L.T., Genova J.L., Rostagno H.S. (2024). Brazilian Tables for Poultry and Swine: Composition of Feedstuffs and Nutritional Requirements.

[7] Rodrigues P.B., Rostagno H.S., Albino L.F.T., Gomes P.C., Barboza W.A., Santana R.T. (2001). Valores Energéticos do Milheto, do Milho e Subprodutos do Milho, Determinados com Frangos de Corte e Galos Adultos. Rev. Bras. Zootecnia.

[8] Brito A.B., Stringhini J.H., Cruz C.P., Xavier S.A.G., Leandro N.S.M., Café M.B. (2005). Efeito do gérmen integral de milho sobre o desempenho e rendimento de carcaça de frangos de corte. Arq. Bras. Med. Veter. Zootecnia.

[9] Brito A.B.D., Stringhini J.H., Xavier S.A.G., Gonzales E., Leandro N.S.M., Café M.B. (2011). Digestibilidade dos aminoácidos do milho, farelo de soja e gérmen integral de milho em galos e frangos de corte cecectomizados. Rev. Bras. Zootecnia.

[10] Adedokun S.A., Utterback P., Parsons C.M., Adeola O., Lilburn M.S., Applegate T.J. (2009). Comparison of endogenous amino acid flow in broilers, laying hens and caecectomised roosters. Br. Poult. Sci..

[11] Sibbald I.R. (1976). A Bioassay for True Metabolizable Energy in Feedingstuffs1. Poult. Sci..

[12] Helrich K.C., AOAC (1990). Official Methods of Analysis of the AOAC.

[13] Hagen S.R., Frost B., Augustin J. (1989). Precolumn Phenylisothiocyanate Derivatization and Liquid Chromatography of Amino Acids in Food. J. Assoc. Off. Anal. Chem..

[14] Eder K. (1995). Gas Review chromatographic analysis of fatty acid methyl esters. J. Chromatogr. B.

[15] Council N.R. (1994). Nutrient Requirements of Poultry: Ninth Revised Edition.

[16] de Blas C., Mateos G.C., Rebollar P.G., Gorrachategui M., Mateos G.G. (2019). Tablas FEDNA de Composición y Valor Nutritivo de Alimentos Para la Fabricación de Piensos Compuestos.

[17] Aguihe P.C., Castelani A.B., Ospina-Rojas C.I., Iyayi E.A., Pozza P.C., Murakami A.E. (2024). Interaction effects of glycine equivalent and standardized ileal digestible threonine in low protein diets for broiler grower chickens. Anim. Biosci..

[18] Fouad A.M., El-Senousey H.K. (2014). Nutritional Factors Affecting Abdominal Fat Deposition in Poultry: A Review. Asian-Australas J. Anim. Sci..

[19] Dale N.M., Fuller H.L. (1986). Repeatability of True Metabolizable Energy Versus Nitrogen Corrected True Metabolizable Energy Values. Poult. Sci..

